# Editorial: Regulators of synapse formation: understanding the molecular mechanisms and its dysregulation in neurodevelopmental disorders

**DOI:** 10.3389/fnmol.2024.1521380

**Published:** 2024-11-22

**Authors:** Takeshi Uemura

**Affiliations:** Division of Gene Research, Research Center for Advanced Science and Technology, Shinshu University, Matsumoto, Japan

**Keywords:** synapse formation, neurodevelopmental disorders, SCN2A, SLITRK3, herbicide glufosinate ammonium, NLGN1, IgSF21, neogenin

Synapse formation in the brain is a highly orchestrated process regulated by a complex interplay of environmental influences, genetic factors, and molecular mechanisms. Disruptions in these regulatory processes can lead to neurodevelopmental disorders, such as autism spectrum disorder (ASD), intellectual disability, and schizophrenia, with profound and lasting impacts on cognitive and behavioral functions. This Research Topic comprises six articles that explore the latest discoveries in the molecular pathways involved in synaptic development and their dysregulation. These articles offer valuable insights into the formation and function of synapses and reveal how genetic and environmental factors can influence these processes. This understanding lays the foundation for the development of novel therapeutic approaches.

The article by Brown et al. investigates how *de novo* mutations in the ASD-associated gene *SCN2A*, which encodes a voltage-gated sodium channel, affect neuronal excitability and synapse formation. The authors utilized iPSC-derived neurons lacking *SCN2A* as well as patient-derived iPSC neurons carrying a *de novo* R607^*^ truncating variant, demonstrating that both complete loss of SCN2A and carrying the R607^*^ mutation reduced synapse formation and excitatory synaptic activity. They also found that neurons with the R607^*^ variant exhibit a loss-of-function effect on neuronal function and development, but this effect is not entirely identical to the complete loss of SCN2A protein. This study offers valuable insights into how *SCN2A* mutations contribute to synaptic dysfunction associated with ASD.

Another study by Efthymiou et al. reports de novo variants in *SLITRK3* (Slit and Trk-like family member 3) identified in three unrelated families affected by epileptic encephalopathy with extensive neurological impairments. SLITRK3, a synaptic cell adhesion molecule, is vital for regulating neurite outgrowth and development of inhibitory synapses. Using heterologous cells and primary cultured hippocampal neurons overexpressing patient-derived *SLITRK3* mutations, they demonstrated that these mutations lead to loss of SLITRK3 function by impairing its transport to the cell membrane surface. Additionally, the analysis of *SLITRK3* knockout mice further underscores the importance of SLITRK3 in inhibitory synaptic organization, providing insights into how inhibitory synaptic dysfunction may contribute to epilepsy and other neurodevelopmental disorders.

In addition to genetic factors, environmental factors are crucial to neurodevelopmental health. The article by Izumi et al. examines the effects of maternal exposure to the herbicide glufosinate ammonium (GLA) on synapse formation in the offspring, emphasizing the potential role of environmental factors in neurodevelopmental disorders. These findings reveal that maternal GLA exposure disrupts typical synaptic development by altering the expression of genes vital for synapse formation, leading to synaptic abnormalities. This study highlights the importance of assessing prenatal environmental risks that may contribute to synaptic pathologies and stresses the need for further studies on how environmental factors influence neurodevelopmental health.

Focusing on synapse organizers and signaling pathways, the articles by Szíber et al. and Chofflet et al. provide significant insights into the molecular mechanisms driving synaptic differentiation at excitatory and inhibitory synapses, respectively. Szíber et al. highlight the molecular processes behind excitatory synapse differentiation, specifically the phosphorylation of neuroligin-1 (NLGN1) at tyrosine residues. This phosphorylation, mediated by receptor tyrosine kinases, such as TrkB, plays a crucial role in recruiting postsynaptic density proteins, which are essential for the formation of the synaptic scaffold. This study identified TrkB as a key kinase for NLGN1 phosphorylation, which subsequently facilitates the assembly of PSD-95 at excitatory synapses. This study provides valuable insights into the molecular specificity mechanisms at excitatory synapses and has potential therapeutic implications for neurodevelopmental disorders related to synapse formation.

Chofflet et al. investigate the immunoglobulin superfamily member 21 (IgSF21)–neurexin2α (Nrxn2α) complex, a critical organizer of inhibitory synapses in the brain, and provided insights into the binding mode of the IgSF21-Nrxn2α complex. Their research revealed distinct signaling pathways by which IgSF21 and neuroligin-2 (NLGN2) regulate the organization of inhibitory synapses, also highlighting differences in compartment-specific roles related to GABAergic presynaptic differentiation. While both IgSF21 and NLGN2 rely on c-Jun N-terminal kinase (JNK) signaling, NLGN2 also requires the activation of CaMKII and Src kinase. These pathway-specific insights enhance our understanding of compartment-specific synapse formation and could inform therapeutic advances in neurodevelopmental disorders.

The article by Sempert et al. reveals a crucial role of neogenin in the development of dendritic spines, which are essential for synaptic plasticity, learning, and memory. The authors showed that neogenin, along with its ligand molecule, Repulsive Guidance Molecule a (RGMa), regulates actin dynamics via the WAVE Regulatory Complex (WRC), promoting spine maturation into stable mushroom-shaped forms necessary for strong synaptic connections. Disruptions in Neogenin or RGMa signaling lead to a reduction in mature spines and an increase in immature spines, underscoring the importance of neogenin-RGMa signaling in synaptic development and plasticity. These findings provide valuable insights into the structural mechanisms underlying memory processes.

